# Current trends in managing TMJ ankylosis in children—summing it up with a case report

**DOI:** 10.1093/jscr/rjac550

**Published:** 2023-02-15

**Authors:** Namdeo Prabhu, Rakhi Issrani, Kiran Kumar Ganji

**Affiliations:** Department of Oral & Maxillofacial Surgery and Diagnostic Sciences, College of Dentistry, Jouf University, Sakaka, Kingdom of Saudi Arabia; Department of Preventive Dentistry, College of Dentistry, Jouf University, Sakaka, Kingdom of Saudi Arabia; Department of Preventive Dentistry, College of Dentistry, Jouf University, Sakaka, Kingdom of Saudi Arabia; Department of Periodontics and Implantology, Sharad Pawar Dental College and Hospital, Datta Meghe Institute of Medical Sciences, Sawangi Meghe, Wardha, Maharashtra, India

## Abstract

Temporomandibular joint (TMJ) ankylosis involves fusion of the mandibular condyle to the base of the skull. The treatment of TMJ ankylosis poses a significant challenge because of technical difficulties and a high incidence of recurrence. The experience of managing one such case is reported in light of a review of the literature on this condition. This report describes a case of a 7-year-old girl with inability to open her mouth, diagnosed with unilateral right bony TMJ ankylosis. Key management principles included adequate removal of the ankylotic mass, gap arthroplasty with interpositional temporalis myofascial grafting and post-operative physiotherapy. The patient maintains a satisfactory maximal incisal opening till the present day. A detailed history, clinical and functional examination, radiographic examination facilitating correct diagnosis followed by immediate surgical intervention, and physiotherapy might be beneficial to restore physical, psychological and emotional health of the child patient with TMJ ankylosis.

## INTRODUCTION

Temporomandibular joint (TMJ) ankylosis is a well-known disorder that is characterized by restriction of mouth opening from partial reduction to complete immobility of jaw [1]. Patients with TMJ ankylosis experience a range of clinical, social, and psychological problems. Just to name a few - mastication, digestion, speech, appearance, and hygiene can have a great impact on personality [2]. It is well documented that malocclusion is a well-known occurrence in these cases [3, 4]. Although voluminous research articles are available, with lot of innovations and ideas but it has added to the never ending confusion of which treatment is the best because recurrence still remains the major problem when treating TMJ ankylosis [5–7]. This paper describes a case of unilateral right bony TMJ ankylosis and its successful management with gap arthroplasty in a 7-year-old child with a brief literature review.

## CASE PRESENTATION

A 7-year-old girl, reported to our hospital with a chief complaint of inability to open her mouth since 2–3 years. History revealed that the patient had a fall when she was 5 years old and had a blow on right side of her face. She had pain and swelling on right TMJ area, which progressively subsided. However, there was also gradual reduction of mouth opening as a result of which she was unable to eat properly.

## CLINICAL EXAMINATION

**Figure 1:**
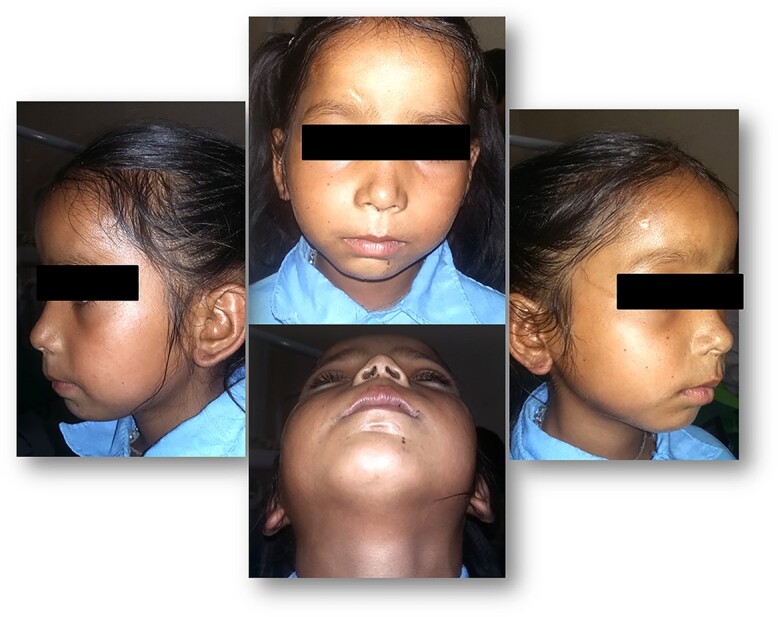
Pre-operative facial profile.

**Figure 2:**
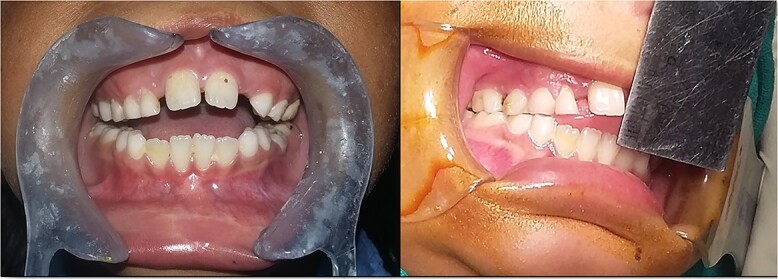
Pre-operative MIO (5 mm).

The clinical examination revealed a hypoplastic mandible with lower facial asymmetry and fullness of cheek on right side. The patient had trismus with a maximal incisal opening (MIO) of 5 mm. There was no palpable movement over the right TMJ and only slight rotation on left side (Figs 1 and 2).

## RADIOGRAPHIC FEATURES

**Figure 3:**
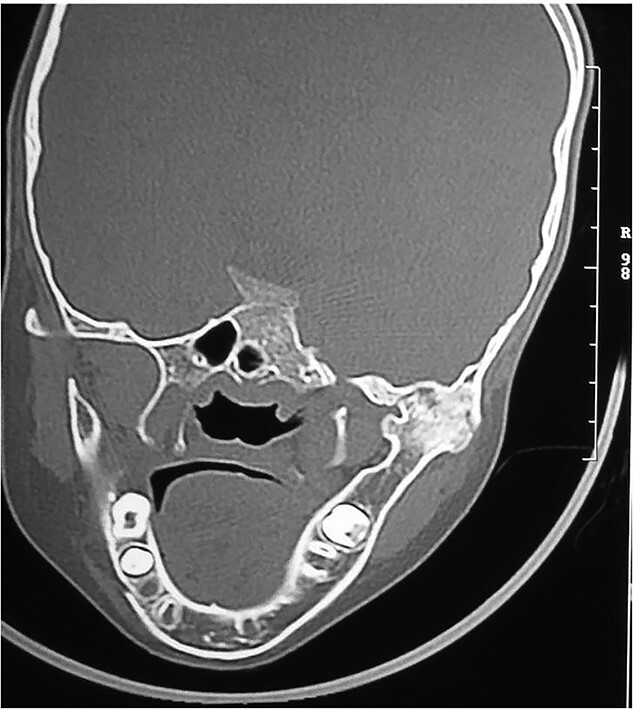
Right side bony ankylosis in coronal section CT.

Orthopantomogram and computed tomography (CT) scan with 3D reconstruction revealed a lack of structural organization and obliteration of right TMJ space. The right body of mandible was arch shaped due to restriction of the right condyle with right coronoid pushing upwards. There was also occlusal cant on frontal CT scan. Coronal view of CT scan demonstrated ankylotic mass. Based on these finding, a diagnosis of unilateral bony ankylosis of TMJ on right side was confirmed (Fig. 3).

## TREATMENT

**Figure 4:**
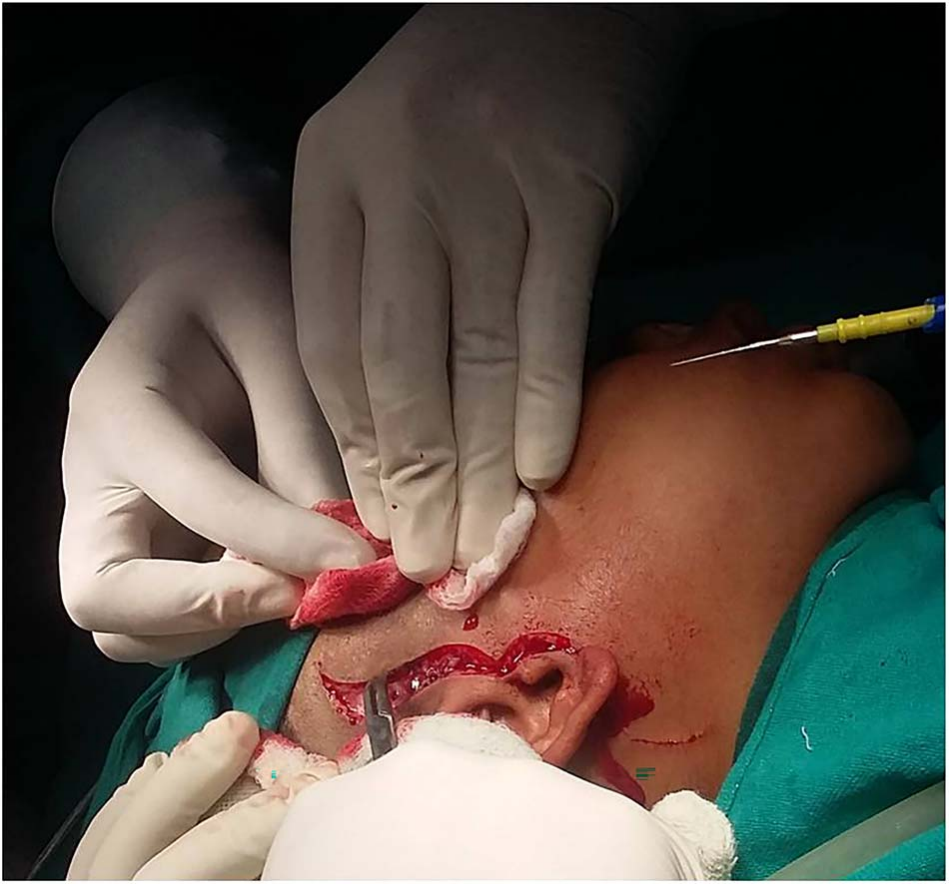
Alkyalam Briyam incision.

**Figure 5:**
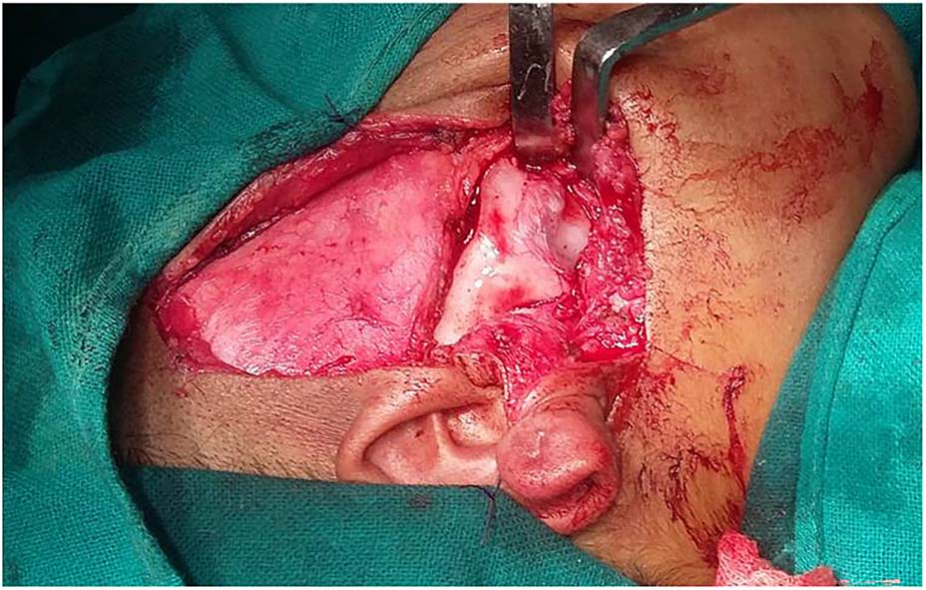
Ankylotic mass.

**Figure 6:**
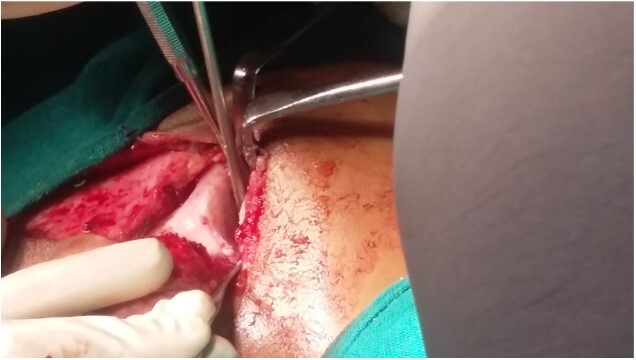
Separation of condylar unit from skull base.

**Figure 7:**
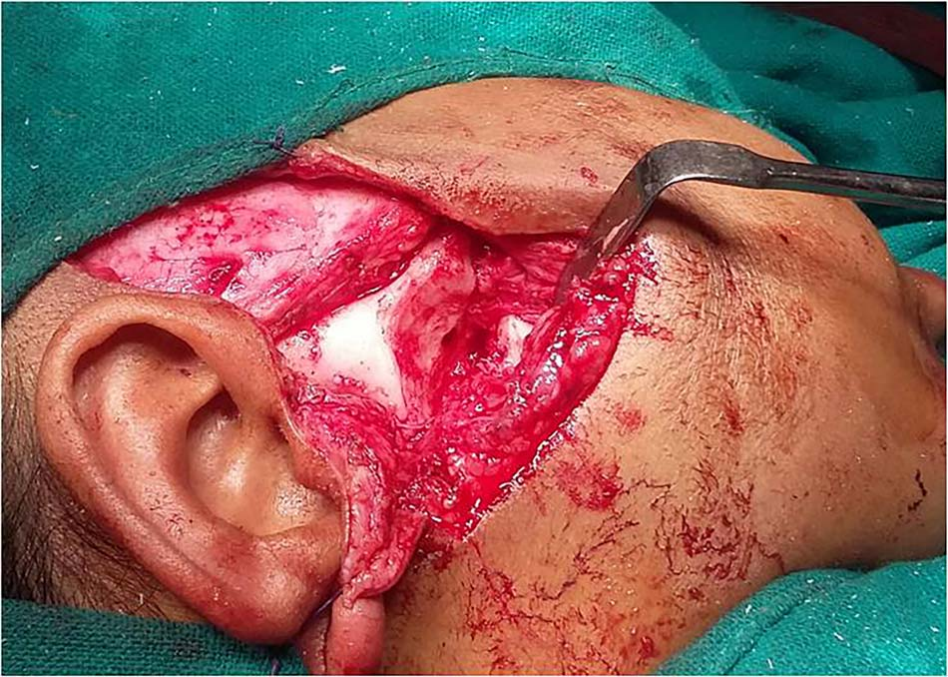
Gap arthroplasty.

**Figure 8:**
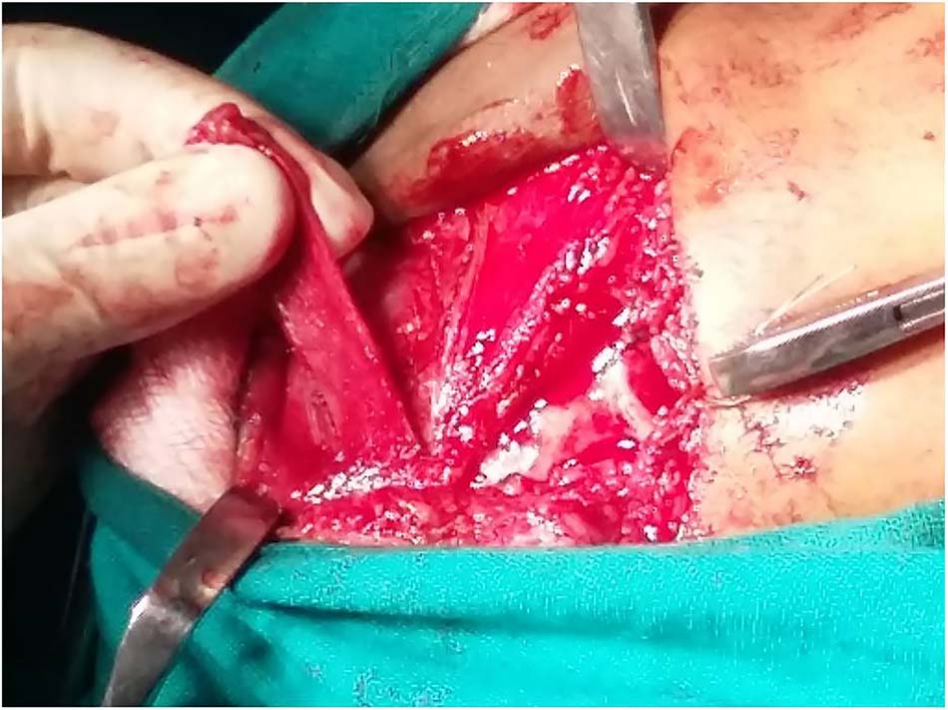
Temporalis myofascial flap for interpositional gap arthroplasty.

A surgical treatment of gap arthroplasty with interpositional temporalis myofascial flap on right TMJ was planned under general anesthesia. In order to approach the TMJ area, a pre-auricular incision was utilized [8, 9]. The incision in shape of big question mark avoiding injury to the superfacial temporal vessels and facial nerve was made till the shining surface of temporalis fascia and dissection preceded in this plane to zygomatic arch and extended anteriorly and posteriorly to expose the limits of the ankylosis. Thereafter periostium over the zygomatic arch and the ramus incised and elevated (Fig. 4). After an adequate exposure of site of bony block, bone was removed by using a round bur until a thin cortical bone was left in the depth. To prevent injury to internal maxillary artery or pterygoid plexus of veins, a malleable retractor and surgical burs at slow speed were used. The joint cavity was then irrigated with betadine and irregular edges of segments were shaved by surgical bur and disconnected completely the ramus from the upper bony block (Figs 5 and 6). In this case, glenoid fossa was recontoured as much as anatomical as possible. A gap of at least 1.5 cm was created between glenoid fossa and mandible followed by insertion of interpositional temporalis myofascial graft (Figs 7 and 8). Zygomatic arch osteotomy was also performed, which immensely eased the coronoidectomy procedure but added to complexity of the surgery and fixation hardware had to be used in addition to fix the arch back into its position.

**Figure 9:**
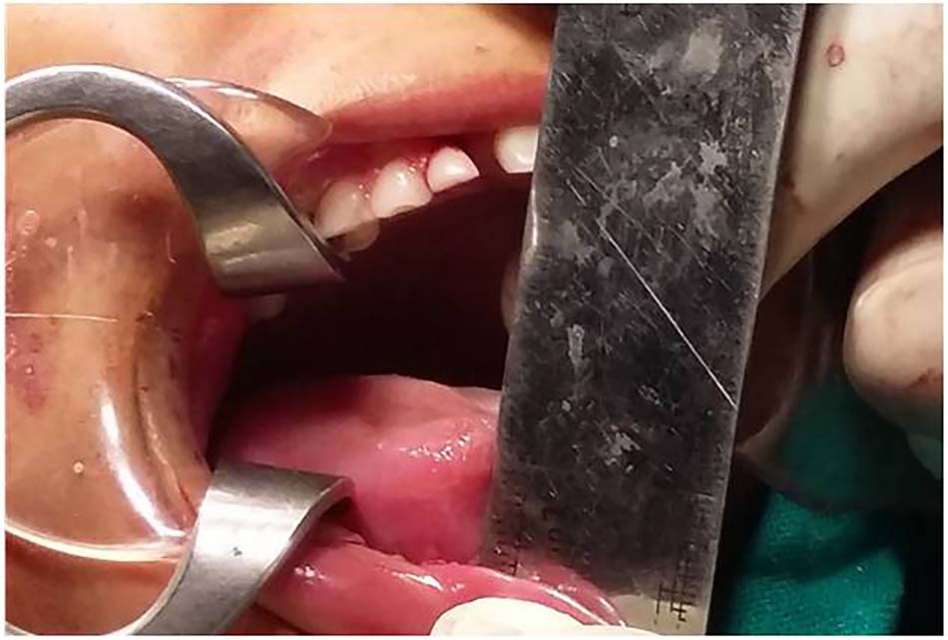
Immediate post-operative clinical picture.

**Figure 10:**
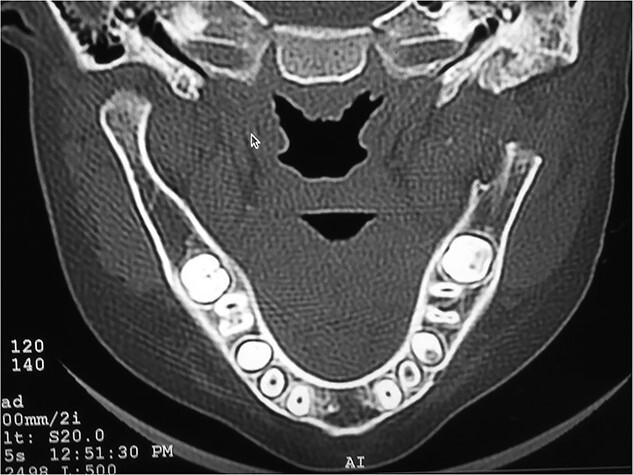
Post-operative coronal section CT image.

Mouth opening of ~37 mm was done using Hister’s jaw opener at time of surgery (Figs 9 and 10). Suction drain was placed, and the flap was sutured using 3–0 vicryl for deeper layers and skin was closed using 4–0 prolene.

## POST-OPERATIVE COURSE

**Figure 11:**
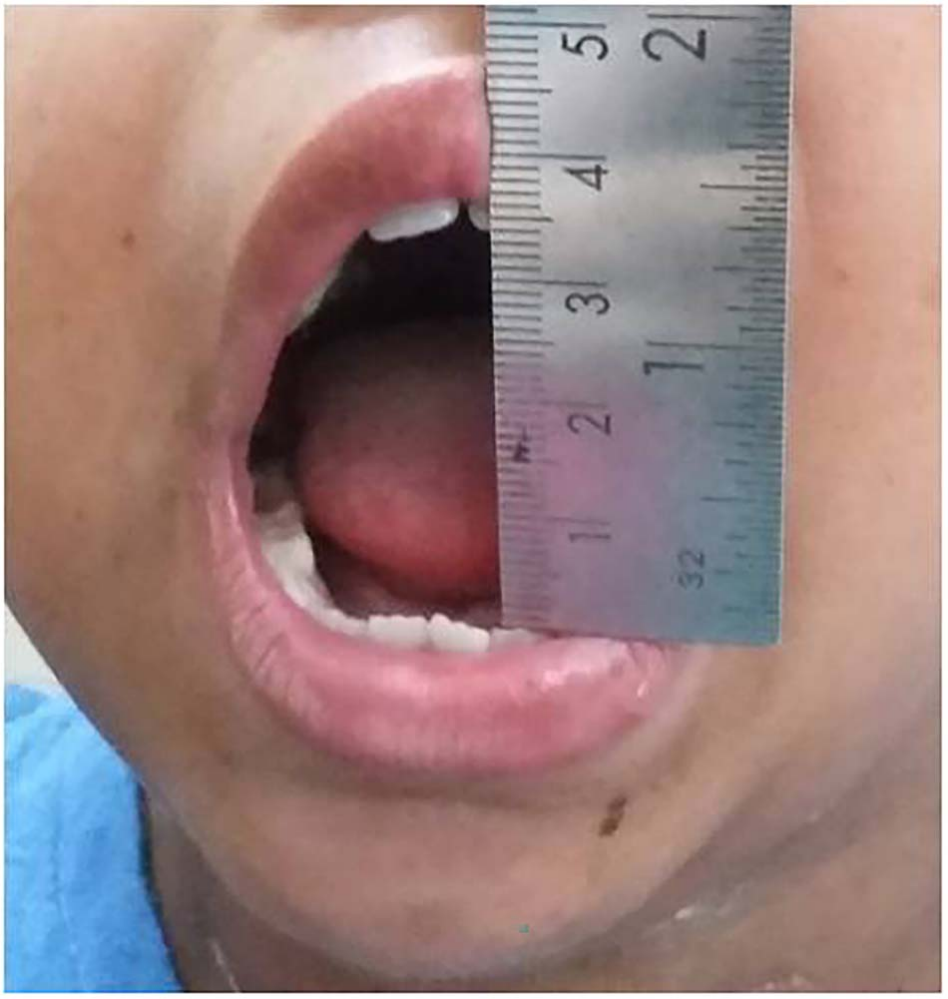
Follow-up after 6 months.

Post-operative course was uneventful. Physiotherapy commenced 3 days post-operatively. Mouth opening exercises were given by using Hister’s mouth gag. The patient was instructed to continue with exercises for at least a period of 1 year. On follow-up after 2 weeks, the mouth opening was found to be 35 mm that was maintained in the next follow-up after 6 months (Fig. 11).

## DISCUSSION

TMJ ankylosis surgery has to be considered in a stepwise approach to avoid any untoward sequelae.

### Etiology

Mandibular hypomobility can be caused by trauma, radiation therapy, surgical removal of TMJ tumors, infection, and systemic diseases [10, 11]. On the other hand, Kaban *et al*. had noticed that infection remains the most common cause of TMJ ankylosis in children. On the contrary, Roychoudhury *et al*. reported trauma as a single most important reason for TMJ ankylosis [2]. A number of systemic diseases have also been cited as contributing factors, including ankylosing spondylitis, rheumatoid arthritis, Paget's disease, pseudo-hypoparathyroidism, psoriasis and burns [12, 13]. In this case, trauma was the most important factor for development of TMJ ankylosis that might be attributed to hilly regions where fall from height is very common and difficult lifestyle of the patients wherein children and young adults have to climb trees for fetching fruits as a source of nutrition. This was further complicated by the issues like severe shortage of healthcare professionals and difficult access to tertiary and quaternary health facilities causing no prompt treatment and ensuing complications.

### Classification

TMJ ankylosis is classified according to Perrott and Kaban [14]:

(i) Location (intra-articular or extra-articular),
(ii) Type of tissue involved (e.g. bone, fibrous or fibro-osseous), and
(iii) Extent of fusion (complete or incomplete).

Classification followed for this case is the one proposed by Sawhney [15] as shown in Table 1.

**Table 1:** Classification of TMJ ankylosis proposed by Sawhney [15]

**Type**	**Description**
Type one	Minimal bony fusion, but extensive fibrous adhesions around the joint
Type two	More bony fusion especially at the outer edge of the articular surface, but no fusion within the more medial area of the joint
Type three	There is a bridge of bone between the mandible and the temporal bone
Type four	The joint is replaced by a mass of bone

### Treatment

A seven-step protocol has been developed as follow [5, 16]:

(i) aggressive resection of ankylotic segment,

(ii) ipsilateral coronoidectomy,

(iii) contralateral coronoidectomy when necessary,

(iv) lining of the joint with temporalis fascia or cartilage,

(v) reconstruction of ramus with a costochondral graft,

(vi) rigid fixation of graft, and

(vii) early mobilization and aggressive physiotherapy.

In the field of maxillofacial surgery, the TMJ arthroplasty is one of the more mysterious operations due to the nature of the problem as well as the fact that several follow-up options are available to the surgeon [17–24].

1. Gap arthroplasty,

2. Interpositional gap arthroplasty, and

3. Joint reconstruction with autogenousor alloplastic materials.

Table 2 shows the previous studies highlighting the treatment options for TMJ ankylosis.

**Table 2:** Various studies highlighting the treatment options for TMJ ankylosis

**Authors/year**	**Highlights of study**	**Results**
Matsuura *et al.*	Studied the functional and anatomic changes after gap arthroplasty by using animal models.	This procedure for TMJ ankylosis did not restore TMJ functionally and histologically to its preexisting state.
Kaban *et al.*	Gap arthroplasty alone gives rise to a gap between the articular cavity and the mandibular ramus.	Advantages of simplicity and short operating time were found. But disadvantage of generating a pseudo-articulation, with shortening of the mandibular ramus and, increase in the risk of recurrence were also observed.
Vasconcelos *et al.*	Reported eight cases of ankylosis (Types I–IV) treated by gap arthroplasty	No recurrence in their series with a follow-up of at least 24 months. Complications such as the development of an open-bite in bilateral cases, premature occlusion on the affected side with contralateral open-bite in unilateral cases and limited mouth opening post-operatively were possible.
Borcbakan	First to use an acrylic condyle in the surgical treatment of TMJ ankylosis.	Inexpensive material that can be produced locally and does not require an additional surgical site. Acrylic does not produce any long-term complications and is well-tolerated. The only disadvantage of the technique is the development of facial asymmetry when it is used in a child, requiring corrective jaw surgery.
McCarthy *et al.*	Distraction osteogenesis has been proposed for the treatment of bony TMJ joint ankylosis.	Distraction osteogenesis offers many advantages over other more traditional surgical techniques. It has been shown that it can shorten the admission and operation time, the risks of surgery and the possibility of relapse. Above all, the direction and amount of bony lengthening can be controlled, and soft tissues as well as hard tissues can be lengthened.

### Follow-up

In immediate follow-up period it is recommended that potential problem with early mobilization is that it may provoke bleeding and create a large hematoma with delayed healing [12]. But the present case was treated with physiotherapy from the first post-operative day to the extent that was acceptable and comfortable to the patient without any severe pain or discomfort. In long-term follow-up, the interincisal opening of the patients to see if patient is showing any signs of recurrence of ankylosis and whether the mandibular growth is being affected in any way was studied [25].

## CONCLUSION

Traumatic injury to the TMJ should be considered as a risk for ankylosis in children. A careful surgical technique and meticulous long-term physiotherapy are considered essential to achieve a satisfactory result.

## Data Availability

The data set used in the paper will be made available on request from Namdeo Prabhu (drpranam@gmail.com).
